# Leptomeningeal Carcinomatosis Secondary to a Gastrointestinal Stromal Tumor

**DOI:** 10.7759/cureus.16212

**Published:** 2021-07-06

**Authors:** Rachel Wlock, Keshav Patel, Madhav Patel, Armand Tanase

**Affiliations:** 1 Neurology, Western Michigan University Homer Stryker M.D. School of Medicine, Kalamazoo, USA; 2 Internal Medicine, University of Illinois College of Medicine at Chicago, Chicago, USA; 3 Neurology, Georgetown University M.D. School of Medicine, Washington, DC, USA; 4 Internal Medicine, Western Michigan University Homer Stryker M.D. School of Medicine, Kalamazoo, USA; 5 Internal Medicine, Ascension Borgess Hospital, Kalamazoo, USA

**Keywords:** leptomeningeal metastasis, gastric tumor, gastrointestinal stromal tumor (gist), leptomeningeal carcinomatosis, leptomeningeal carcinoma

## Abstract

A 66-year-old male presented to the emergency department (ED) with altered mental status and concern for stroke. Seven months prior to presentation, the patient was diagnosed with a benign gastrointestinal stromal tumor (GIST), for which he did not receive further workup. Initially, there was a concern for a stroke, however, CT of the brain, CT angiography of the head and neck, and MRI of the brain were negative for any acute abnormalities. Lumbar puncture (LP) revealed a low glucose level with increased protein and white blood cells in the cerebrospinal fluid (CSF). The patient did not display the typical signs and symptoms of meningitis, however, inpatient antibiotic treatment was initiated. The patient continued to deteriorate and repeat LP with cytology was ordered, which revealed malignant cells that were similar to the biopsy of the GIST; our patient was diagnosed with leptomeningeal carcinomatosis (LMC) secondary to the GIST. This case report presents a rare complication of a solid GIST and highlights the need for a high clinical index of suspicion for LMC, including those previously deemed to be benign.

## Introduction

Leptomeningeal carcinomatosis (LMC) is a rare complication of metastatic cancer in which cancer spreads to the arachnoid and pia mater. In patients with solid tumors, LMC most commonly occurs with breast cancer, lung cancer, melanoma, and gastrointestinal malignancies [1]. Malignant cells can travel in the subarachnoid space and spread to other locations via the cerebrospinal fluid (CSF). LMC often presents with multifocal neurologic signs and symptoms, which can develop over days to weeks. Characteristic findings include increased intracranial pressure from mass effect or outflow obstruction, cranial nerve or spinal root dysfunction, diffuse cerebral dysfunction, and cerebral edema [2]. In one study, patients with solid tumor LMC most commonly presented with headache (39%), nausea and vomiting (25%), leg weakness (21%), cerebellar dysfunction (17%), altered mental status (16%), diplopia (14%), and facial weakness (13%) [2]. If LMC is suspected, neuroimaging should be pursued. Brain MRI with contrast is the most sensitive diagnostic modality, however, CSF cytology is more specific. MRI findings may include diffuse enhancement of the leptomeninges along the brain sulci or multiple nodules within the subarachnoid space. The most definitive finding is malignant cells in the CSF, but other findings include increased protein, low glucose, or increased lymphocytes [3]. LMC is a rare complication that is difficult to diagnose. In this case study, we discuss a patient who was diagnosed with LMC secondary to a primary solid gastrointestinal stromal tumor (GIST).

## Case presentation

A 66-year-old male with a past medical history of tobacco use, schizophrenia, and recently identified mesenteric mass presented with confusion, weakness, and concern for a possible stroke. At baseline, the patient was ambulatory without neurologic deficits. The 5.4 cm x 5.3 cm mesenteric mass was biopsied seven months prior to presentation and histopathology indicated a benign GIST with a few mitotic figures and the patient did not receive further workup.

Five months prior to this presentation, the patient was brought to the emergency department (ED) with similar symptoms. At that time, it was thought that he had overdosed on his psychiatric medications, which included quetiapine, lithium, and haloperidol. At the current presentation, the patient’s caregiver denied the possibility of overmedication as she kept his medications in a lockbox and personally dispensed them.

CT of the brain, CT angiography of the head and neck, and MRI of the brain with and without gadolinium contrast were negative for any acute abnormalities. Electroencephalogram revealed diffuse slowing, indicating nonspecific encephalopathy.

Lumbar puncture (LP) results (Table 1) revealed colorless CSF with 149 mg/dL protein (normal range 15-45 mg/dL), 8 mg/dL glucose (normal range 40-70 mg/dL), and 65 white blood cells (WBC)/µL (normal range 0-5 WBC/µL). CSF Gram stain did not reveal any organisms and CSF culture was negative. CSF acid fast bacilli culture and smear were negative. The bacterial antigen testing in the CSF was negative for Hemophilus influenzae, *Neisseria meningitdis*, *Streptococcus pneumoniae*, and Group B Streptococcus. CSF fungal culture was negative. CSF microbiology testing was negative for *Cryptococcus neoformans*, cytomegalovirus, Enterovirus, *Escherichia coli*, Hemophilus influenzae, herpes simplex 1 and 2, human herpesvirus 6, human parechovirus, listeria monocytogenes, *N. meningitidis*, *Streptococcus agalactiae*, *S. pneumoniae*, and Varicella zoster. CSF cytology did not show any signs of malignancy though there were some atypical monocytes/macrophages, which was concerning for bacterial meningitis. Both infectious disease and neurology had been consulted and although he did not display the typical signs and symptoms of bacterial meningitis, he was started on ceftriaxone.  

**Table 1:** Cerebrospinal fluid results. LP, lumbar puncture; RBCs, red blood cells; WBCs, white blood cells

	Initial LP	Repeat LP	Normal range
Color	Colorless	Colorless	NA
Description	Clear	Slightly cloudy	NA
RBCs (per μL)	2-3	1,217-1,510	0
WBCs (per μL)	61-65	52-65	0-5
Glucose (mg/dL)	8	25	40-70
Protein (mg/dL)	149	71	15-45

The patient continued to deteriorate and progressed into a comatose state. Repeat brain and spine MRI with and without contrast were negative. The patient underwent a repeat LP (Table 1), which showed 71 mg/dL protein, 25 mg/dL glucose, and 50-60 WBC/µL. The CSF Gram stain, culture, bacterial antigen, and microbiology testing were all negative. Of note, the repeat LP was traumatic and, therefore, 1,217-1,510 red blood cells (RBCs)/µL were present.

**Figure 1:**
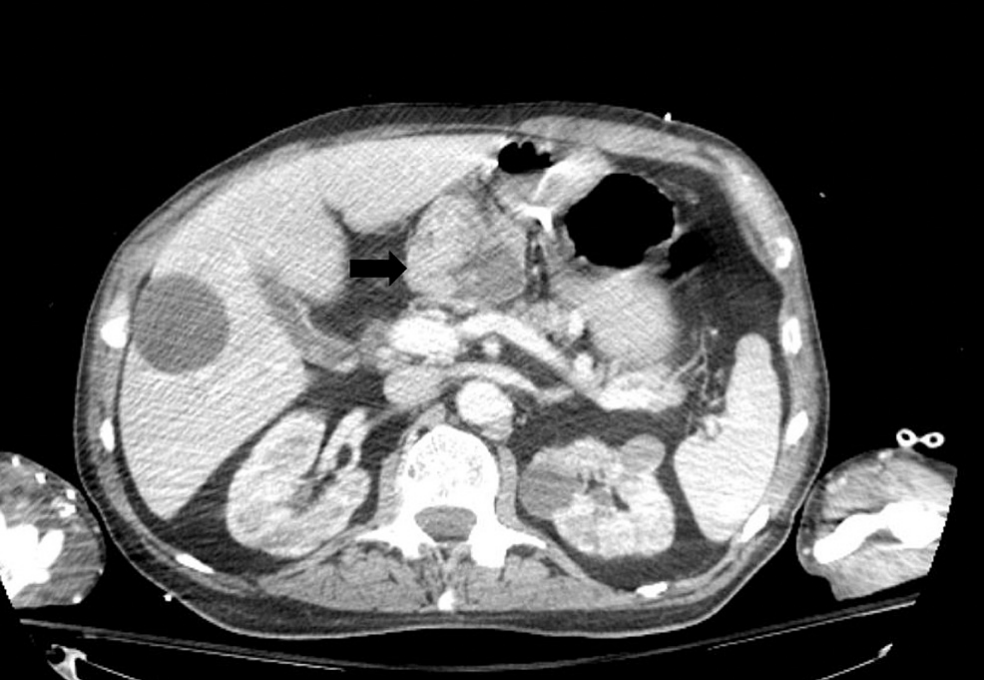
Exophytic solid and cystic gastric mass. Axial CT with contrast revealed a mixed solid and cystic 6.2 cm x 6.1 cm exophytic gastric mass (black arrow) that was located distal to the stomach. This mass was previously determined to be a GIST by biopsy. CT also revealed low-attenuation lesions in the liver and spleen, which were most likely cysts, and multiple small renal cysts along with heterogenous attenuation with a nodular surface, which was most likely secondary to chronic scarring. GIST, gastrointestinal stromal tumor

The CSF cytology was highly suspicious for malignancy with similarity to the neoplastic cells from the mesenteric biopsy seven months ago. At this point, it was determined that the patient's clinical scenario was more likely due to LMC rather than meningitis. Of note, the patient underwent CT chest/abdomen/pelvis during this hospitalization, which revealed a 6.2 cm x 6.1 cm exophytic gastric mass (Figure 1), which was previously determined to be a benign GIST by biopsy. The patient’s GIST was previously measured to be 5.4 cm x 5.3 cm 10 months ago. LMC was not highly suspected at first due to the fact that the GIST was deemed benign and the first LP was not definitively malignant. Due to the poor prognosis, the family decided to move the patient to hospice with comfort measures only. He ultimately died 16 days after his initial presentation to the emergency room.

## Discussion

Leptomeningeal carcinomatosis occurs in 5%-8% of cases of solid tumors [4]. As stated previously, there is no single, uniform presentation that would suggest its presence though there may be multifocal neurologic signs and symptoms, therefore, diagnosis is difficult and requires a high index of suspicion.

The primary tumor which ultimately caused the LMC in this patient was a GIST. Current recommendations regarding GISTs are based on the Joensuu risk stratification, which takes into account tumor size, mitotic index, and primary tumor location [5]. GISTs of any classification have been observed to display malignant behavior given enough time, thus it is not recommended to designate these tumors as benign, but rather to consider the risk of malignancy [6-7]. If possible, the best option to treat a GIST is surgical resection. This is typically considered when the tumor is primary and has not metastasized, especially if it is >2 cm. If metastasis has occurred or the tumor is unable to be resected, imatinib is the treatment of choice. After resection, patients should have a routine follow-up, although there are no guidelines for what is considered an optimal timeline [8-10]. In this case, the patient was confirmed to have a GIST by biopsy and histopathology. The tumor was deemed benign and the patient did not undergo further workup. However, according to current guidelines, this 5.4 cm x 5.3 cm mass should have been excised.

Once LMC is suspected, it is important to confirm the diagnosis as early as possible. The initial evaluation is usually with gadolinium-enhanced brain and spine MRI and CSF studies [11]. MRI will typically show enhancement of the leptomeninges following the lines of the sulci, either focally or diffusely. Additionally, it is helpful to perform the MRI before the LP as the LP can create artifacts in the form of dural enhancement. Experienced neuro-radiologists may or may not be comfortable in differentiating this artifact from the true enhancement of the leptomeninges. In this case, our patient did have an MRI performed prior to the LP. The MRI still showed diffuse sulcal fluid-attenuated inversion recovery (FLAIR) hyperintensity, but this was attributed to medication artifact. CSF cytology may reveal malignant cells and, if so, is the gold standard for diagnosis; however, this should not be performed in patients with large intracranial or spinal masses due to the risk of herniation. False-negative results are known to occur up to 36% of the time, as was the case with the first LP in our patient, and this occurs more commonly with delays in processing and refrigeration [4]. Current recommendations for CSF analysis include obtaining > 10 mL of CSF, processing within 30 min, and avoiding hemorrhagic contamination [12-13]. If initial cytology is negative for malignant cells and clinical suspicion remains high, studies should be repeated. In this patient, two LPs were performed with CSF cytology. Both had atypical monocytes/macrophages, however, only the second one was read as malignant after comparison with the tumor biopsy. For this reason, we emphasize the importance of comparison to prior results, even when pathology is not clearly malignant.

The prognosis of LMC is very poor. Overall survival is about two to four months with treatment and four to six weeks without treatment [11]. Prognosis depends on several factors, including primary tumor type, neurologic deficits, and Karnofsky performance status (KPS) [14]. Treatment of leptomeningeal metastases includes radiation, however, solid tumors do not respond as well as hematologic tumors. Intrathecal and systemic chemotherapy are also employed, most often methotrexate, cytarabine, and thiotepa. Unfortunately, due to the rarity of this illness, data from clinical trials are limited. Other novel therapies are currently under investigation, including monoclonal antibodies such as pembrolizumab and ipilimumab/nivolumab, which are typically employed based on the nature of the underlying malignancy [14-15]. Although these treatments are promising for patients with LMC, our patient had already had a significant decline in functional status and the family did not wish to pursue aggressive therapy any longer, and thus chose to seek hospice care for maximal comfort.

## Conclusions

It is unclear how long this patient had LMC. Five months prior to this presentation, he presented with similar symptoms and his changes in mental status were attributed to an overdose of his psychiatric medications. It is also possible that his changes in mental status were actually secondary to LMC. As this is such a rare presentation of a GIST, it is a difficult diagnosis to make and may require additional thoughtfulness on the part of the clinician. However, through this report, we hope to encourage more providers to obtain a broad differential diagnosis and maintain a high clinical index of suspicion for LMC in patients with neurological deficits and previous history of a benign or malignant tumor. 
